# Romidepsin for the treatment of relapsed/refractory peripheral T cell lymphoma: prolonged stable disease provides clinical benefits for patients in the pivotal trial

**DOI:** 10.1186/s13045-016-0243-8

**Published:** 2016-03-10

**Authors:** Francine Foss, Steven Horwitz, Barbara Pro, H. Miles Prince, Lubomir Sokol, Barbara Balser, Julie Wolfson, Bertrand Coiffier

**Affiliations:** Yale Cancer Center, PO Box 208028, 333 Cedar St, TMP 3, New Haven, CT 06520-8028 USA; Memorial Sloan-Kettering Cancer Center, New York, NY USA; Kimmel Cancer Center, Thomas Jefferson University, Philadelphia, PA USA; Peter MacCallum Cancer Centre, University of Melbourne, Melbourne, Australia; Moffitt Cancer Center, Tampa, FL USA; Veristat, LLC, Southborough, MA USA; Hospices Civils de Lyon, Lyon, France

**Keywords:** Romidepsin, Peripheral T cell lymphoma, Stable disease

## Abstract

**Background:**

Achievement of durable responses in patients with relapsed/refractory peripheral T cell lymphoma (PTCL) is challenging with current therapies, and there are few data regarding the potential benefits of continuing treatment in patients with the best response of stable disease (SD). Histone deacetylase inhibitors are a novel class of drugs with activity in T cell malignancies. Romidepsin was approved by the US Food and Drug Administration for the treatment of relapsed/refractory PTCL based on a pivotal trial demonstrating an objective response rate of 25 % (33/130), including 15 % with confirmed/unconfirmed complete response and a median duration of response of 28 months. Our objective was to further study the clinical benefits of romidepsin in patients that had the best response of SD.

**Methods:**

Patients with PTCL relapsed/refractory to ≥1 prior therapy were treated with the approved dose of 14 mg/m^2^ romidepsin on days 1, 8, and 15 of six 28-day cycles; patients with SD or response after cycle 6 were allowed to continue on study until progression. By protocol amendment, patients treated for ≥12 cycles could receive maintenance dosing twice per cycle; after cycle 24, dosing could be further reduced to once per cycle in those who had received maintenance dosing for ≥6 months.

**Results:**

Of the 32 patients (25 %) with the best response of SD, 22 had SD for ≥90 days (SD90; cycle 4 response assessment). The longest SD was >3 years in a patient who received maintenance dosing of 14 mg/m^2^ on days 1 and 15 beginning in cycle 13. Patients with the best response of SD90 or partial response achieved similar overall and progression-free survival. Prolonged dosing of romidepsin was well tolerated.

**Conclusions:**

We concluded that patients who achieve SD may consider continuing treatment because the clinical benefits of romidepsin may extend beyond objective responses.

**Trial registration:**

NCT00426764

## Background

Peripheral T cell lymphoma (PTCL) is a heterogeneous group of aggressive T cell and natural killer (NK)-cell disorders typically associated with poor prognosis [1, 2]. Overall, the known subtypes of PTCL comprise ≈5–10 % of the estimated 71,850 cases of non-Hodgkin lymphoma (NHL) diagnosed in the USA in 2015 [2–4]. The median age has been reported as 62 years, and the most common PTCL subtypes in North America are PTCL not otherwise specified (NOS), angioimmunoblastic TCL (AITL), and anaplastic large cell lymphoma (ALCL); ALCL is divided into those positive or negative for anaplastic lymphoma kinase 1 (ALK-1) [4]. Patients with ALK-1-positive ALCL, who tend to be substantially younger than patients with other subtypes (median age of 34 years) [5], generally have improved prognosis compared with other subtypes [4]. Across all subtypes, increased age is a negative prognostic factor for survival [4, 6].

Most patients with PTCL receive induction chemotherapy (e.g., cyclophosphamide, doxorubicin, vincristine, prednisone (CHOP)) as first-line treatment; however, many patients who respond experience rapid relapse [1, 2, 4, 7, 8]. The use of these anthracycline-based therapies to treat PTCL is a result of successful treatment of B cell lymphomas, [1, 2, 7] although only patients with ALK-1-positive ALCL typically have a favorable prognosis [4, 8]. Accordingly, National Cooperative Cancer Network (NCCN) guidelines for patients with ALK-1-positive ALCL recommend first-line treatment with CHOP or CHOEP [8]. Although there are currently no ALK inhibitors approved for ALK-1-positive ALCL, several are undergoing investigation [9, 10]. For patients with other PTCL subtypes, promising results have been observed in early clinical studies of first-line treatments that combine anthracycline-based chemotherapy regimens with novel agents (romidepsin, brentuximab vedotin, belinostat) [11–13].

According to the NCCN guidelines for PTCL, patients with relapsed/refractory disease are those with less than complete response (CR) or loss of CR to first-line therapy [8]. For patients with relapsed/refractory PTCL, NCCN guidelines group those with CR or partial response (PR) and consider stable disease (SD) or progressive disease (PD) as a lack of response to treatment and a trigger to switch therapy [8].

Achievement of durable responses in patients with relapsed/refractory PTCL is difficult, and there are few treatment options [2, 4, 14]. Additionally, a retrospective analysis of patients with PTCL (*N* = 205) demonstrated that objective response rates (ORRs) and progression-free survival (PFS) decrease with each line of therapy [15]. Thus, in the setting of relapsed/refractory PTCL, overall clinical benefit and not just achievement of an objective response must be carefully considered when initiating, continuing, stopping, or switching therapies. There are few data regarding the potential benefits of continuing therapy for patients with relapsed/refractory PTCL with best response of SD.

Romidepsin is a histone deacetylase inhibitor approved by the US Food and Drug Administration for the treatment of cutaneous TCL (CTCL) in patients who have received at least one prior systemic therapy and PTCL in patients who have received at least one prior therapy [16]. Approval in PTCL was primarily based on results from the pivotal phase 2, single-arm, open-label study in patients with relapsed/refractory PTCL (*N* = 131) [16–18]. This pivotal study demonstrated an ORR of 25 % including 15 % with confirmed/unconfirmed CR (CR/CRu) [16–18] and a median duration of response (DOR) of 28 months (median follow-up 22.3 months) [18] with the longest response ongoing at 56 months [19]. While achievement of CR/CRu was associated with prolonged survival vs all other outcomes, patients who achieved PR or SD for ≥90 days (SD90) had similar long-term outcomes, and the majority of those with best response of SD had SD90 [18]. The objective of the analyses reported herein was to further examine the clinical benefit of SD in patients with relapsed/refractory PTCL treated with romidepsin in the pivotal study.

## Results

### Patient characteristics and disposition

Of the 130 patients with histologically confirmed PTCL, 32 (25 %) had the best response of SD. Baseline patient characteristics among those with best response of SD were similar to those of the overall population (Table 1). The majority of patients (overall or with best response of SD) had advanced disease (stage III/IV, International Prognostic Index ≥2, Eastern Cooperative Oncology Group (ECOG) 1 to 2) and had received ≥2 prior therapies for PTCL. Most discontinuations occurred during cycles 1 to 2 of treatment; 59 patients (45 %) were treated for ≥3 cycles. The majority of patients with best response ≥SD by independent review committee (IRC) assessment who discontinued during cycles 1 and 2 did so due to PD as assessed by the investigator (two patients due to adverse events (AEs)). Twenty-four patients (18 %) received treatment beyond 6 cycles, 18 of whom (14 %) were treated for ≥12 cycles. Romidepsin dose delays and reductions were most common during cycle 2 (19 and 9 %, respectively) and cycle 3 (15 and 7 %, respectively) of treatment.

**Table 1:** Key baseline patient demographics and characteristics

Characteristic	Overall (*N* = 130)^a^	Patients with best response of SD (*n* = 32)
Age, median (range), y	61 (20–83)	61.5 (24–79)
ECOG performance status^b^		
0	46 (35)	10 (31)
1	66 (51)	17 (53)
2	17 (13)	5 (16)
International Prognostic Index, *n* (%)		
<2	31 (24)	7 (22)
≥2	99 (76)	25 (78)
PTCL subtype based on central review, *n* (%)		
PTCL-NOS	69 (53)	16 (50)
AITL	27 (21)	8 (25)
ALK-1-negative ALCL	21 (16)	5 (16)
Other	13 (10)c	3 (9)^d^
Stage III/IV at diagnosis, *n* (%)	91 (70)	20 (63)
Number of prior therapies, *n* (%)		
1	38 (29)	13 (41)
2	44 (34)	11 (34)
3	19 (15)	3 (9)
4	15 (12)	4 (13)
>4	14 (11)	1 (3)
Type of prior therapy, *n* (%)		
Chemotherapy	129 (99)	31 (97)
Monoclonal antibody therapy	20 (15)	3 (9)
Other immunotherapy	14 (11)	1 (3)
Radiation	31 (24)	8 (25)
ASCT	21 (16)	3 (9)
Refractory to most recent therapy, *n* (%)	49 (38)	8 (25)

*ASCT* autologous stem cell transplant

^a^ PTCL histologically confirmed by central pathology review

^b^ One patient in the overall population had missing ECOG performance status at baseline

^c^Includes enteropathy-associated TCL (6), subcutaneous panniculitis-type TCL (3), ALK-1-positive ALCL (1), cutaneous γδ TCL (1), extranodal NK/TCL nasal type (1), and transformed mycosis fungoides (1)

^d^Includes enteropathy-type TCL (1), subcutaneous panniculitis-like TCL (1), and cutaneous γδ TCL (1)

### Long-term outcomes

**Fig. 1:**
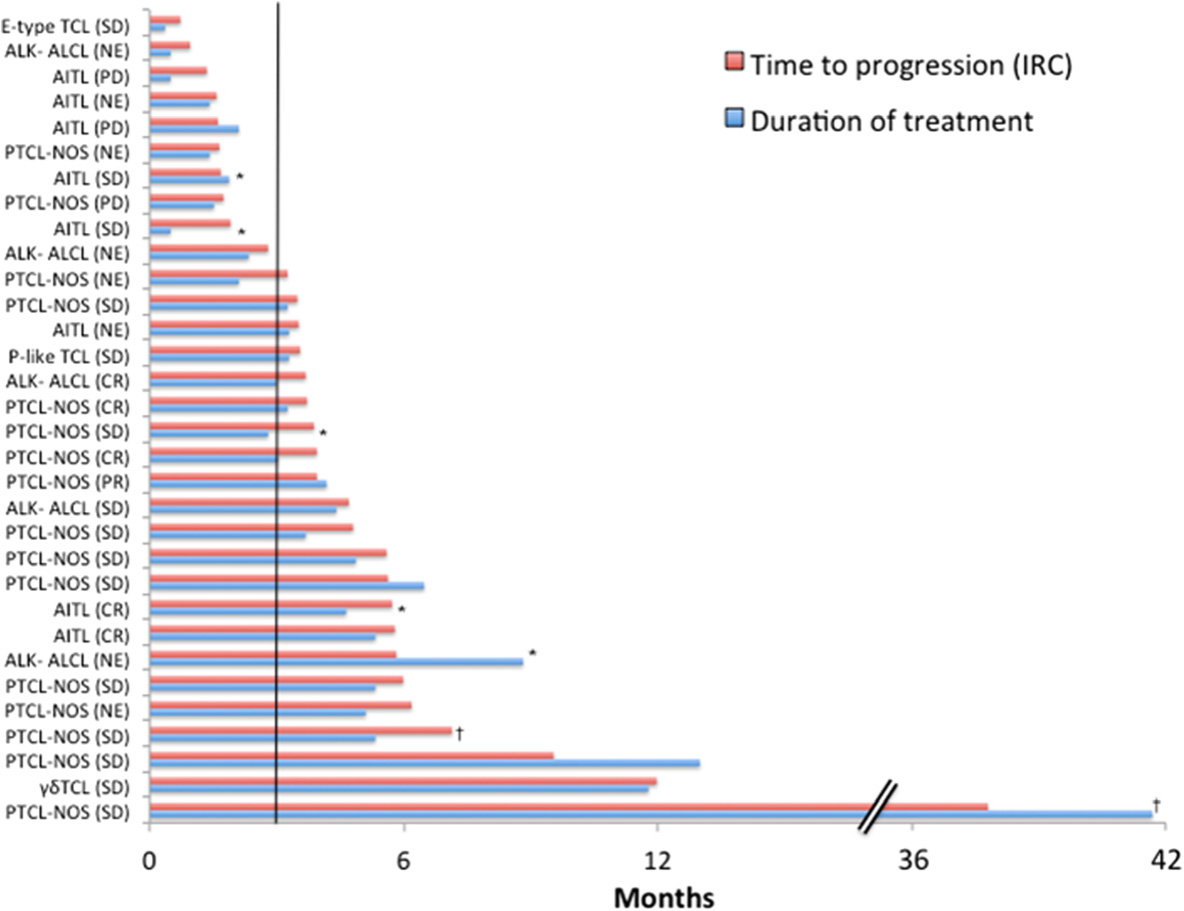
TTP and duration of treatment in patients with best response of SD to romidepsin. PTCL subtypes and response by exploratory PET endpoint are shown on *Y* axis. *Vertical line* indicates 90 days of treatment; *asterisk* indicates patients that discontinued due to adverse event; and *dagger sign* indicates patients that discontinued due to patient decision. *E-type TCL* enteropathy-type T cell lymphoma, *P-like TCL* subcutaneous panniculitis-like T cell lymphoma

Of the 32 patients with the best response of SD, 22 (69 %) had SD90 including 14 of 16 patients with PTCL-NOS, 3 of 8 patients with AITL, 3 of 5 patients with ALK-1-negative ALCL, and 2 of 3 patients with rare subtypes (subcutaneous panniculitis-like TCL and cutaneous γδ TCL; Fig. 1). Thus, for the most common subtypes, the rate of disease control (CR/CRu + PR + SD90) was 49 % (34/69) for PTCL-NOS, 44 % (12/27) for AITL, and 38 % (8/21) for ALK-1-negative ALCL. Six patients had SD for ≥6 months, and the longest SD was > 3 years in duration (Fig. 1). Of note, 6 patients with best response of SD (IRC) were found to have a response (5 CR, 1 PR) by exploratory PET endpoint. All 6 of these patients had achieved SD90 by IRC assessment (time to progression (TTP) 112–176 days). For the 6 patients with SD (IRC) for ≥6 months, 5 also had SD by PET endpoint; 1 did not have PET assessments (Fig. 1). Three patients with best response of SD (IRC) were found to have PD by exploratory PET endpoint, each with a short TTP (IRC) of 41, 49, and 53 days.

**Fig. 2:**
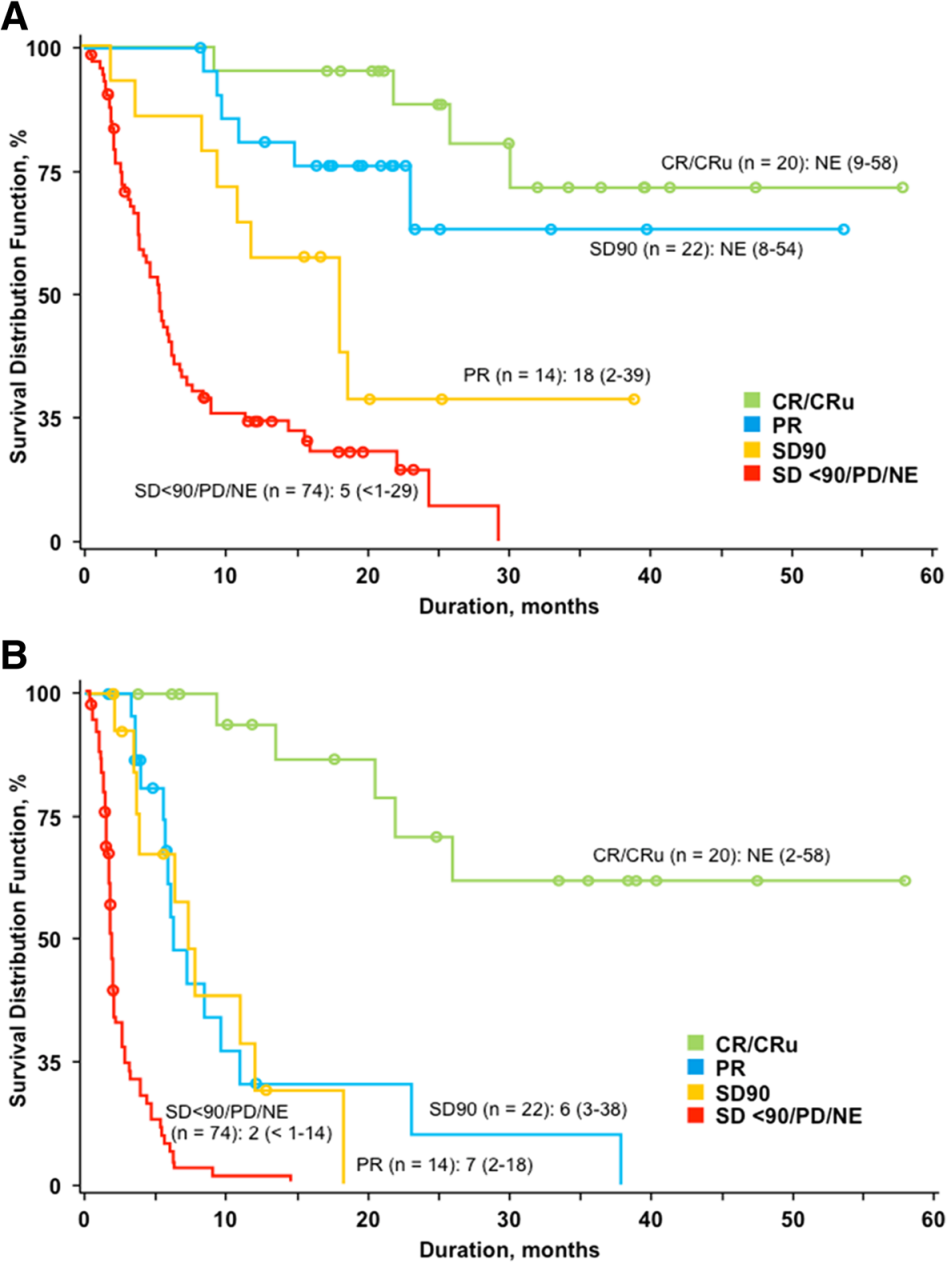
Survival based on clinical IRC assessment by best response to romidepsin (*n* = 130). Progression-free survival (**a**) and overall survival (**b**). Patients with insufficient efficacy data to determine response due to early termination (NE; *n* = 29) were included as nonresponders. *NE* not evaluable

As previously reported with an earlier data cutoff (December 31, 2011) [18], for patients with the best response of SD90, PFS and OS were not statistically different compared with patients who achieved PR (Fig. 2). The patient with the longest reported SD (>3 years) was a 61-year-old woman with stage III PTCL-NOS at diagnosis and ECOG performance status of 1. Prior systemic therapies included CHOP and oral cyclophosphamide, etoposide, and cisplatin. The only drug-related event reported was grade 3 vomiting during cycle 22, which resolved with prochlorperazine in 2 days and did not result in dose changes. Beginning at cycle 13, romidepsin was administered on an every-other-week schedule (days 1 and 15 of 28-day cycles). This patient discontinued at her request after 42 months of treatment. Upon review by the IRC, she was determined to have PD at 38 months of treatment, 4 months before she decided to discontinue. The patient with the second longest reported SD (≈12 months) was a 60-year-old man with stage I cutaneous γδ TCL at diagnosis. Prior therapy included alemtuzumab. Possibly drug-related AEs included grade 3 pyrexia and cellulitis during cycle 6 (history of cellulitis prior to trial) and grade 4 neutropenia during cycle 12. He had a dose withheld in cycle 12 due to infection, pyrexia, and neutropenia and discontinued romidepsin due to PD after 12 months of treatment. At the latest follow-up, all patients with best response of SD had discontinued romidepsin treatment: 24 (75 %) due to PD, 5 (16 %) due to AEs, 2 (6 %) due to patient decision, and 1 (3 %) due to insufficient response.

### Toxicity

**Fig. 3:**
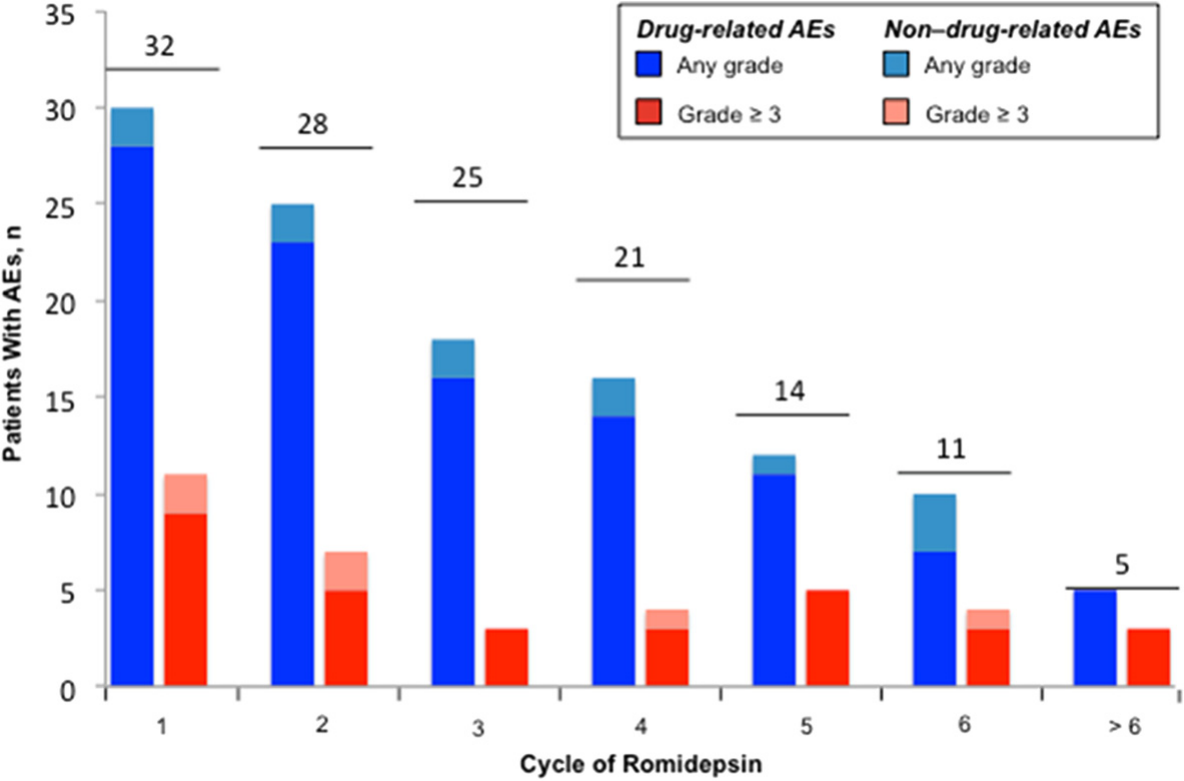
Patients with AEs by cycle in patients with best response of SD to romidepsin. *Numbered bars* represent the number of patients with SD treated in each cycle. Those who experienced drug-related AEs may have also experienced non-drug-related AEs

Similar to what was reported for the overall population [17], the most commonly reported any grade AEs in patients with best response of SD were nausea, asthenia/fatigue, infections (all types pooled), and dysgeusia (Table 2). Nearly all patients with best response of SD experienced ≥1 AE (31/32 (97 %)); this included 30 (94 %) with drug-related AEs, 23 (72 %) with grade ≥3 AEs, and 19 (59 %) with grade ≥3 drug-related AEs. Neutropenia, thrombocytopenia, infections (all types pooled), and anemia were the only grade ≥3 AEs reported in >1 patient with best response of SD. Incidence of any grade drug-related AEs was highest during cycle 1. Patients with best response of SD to romidepsin experienced AEs throughout their treatment; though, notably, all patients with AEs reported after cycle 6 had ≥1 drug-related AE, and non-drug-related grade ≥3 AEs occurred rarely after cycle 2 (Fig. 3).

**Table 2:** Most common AEs by treatment cycle in patients with best response of SD to romidepsin

Drug related/non-drug related, *n*	Treatment cycle							
	Any (*n* = 32)	1 (*n* = 32)	2 (*n* = 28)	3 (*n* = 25)	4 (*n* = 21)	5 (*n* = 14)	6 (*n* = 11)	>6 (*n* = 5)
Any grade AEs reported in > 20 % of patients with best response of SD								
Nausea	22/2	18/2	6/0	5/0	5/1	5/0	0/0	1/0
Asthenia/fatigue	19/1	13/0	6/0	4/0	4/0	3/1	2/1	1/0
Infections SOC	6/11	4/6	1/3	1/2	0/5	1/1	1/2	1/2
Dysgeusia	14/0	8/0	7/0	1/0	2/0	0/0	0/0	0/0
Vomiting	12/1	5/0	4/1	4/0	3/1	4/0	0/0	1/0
Diarrhea	7/5	5/1	4/2	1/0	1/0	0/1	0/1	1/0
Constipation	5/6	3/3	0/0	2/0	0/0	1/1	0/0	0/1
Anorexia	11/0	6/0	3/0	3/0	1/0	2/0	0/0	1/0
Thrombocytopenia	10/0	6/0	2/0	3/0	2/0	2/0	2/0	0/0
Pyrexia	7/1	1/1	1/0	0/1	2/1	1/1	1/1	1/1
Neutropenia	7/0	4/0	4/0	3/0	2/0	1/0	1/0	2/0
Anemia	6/1	1/1	2/0	1/0	1/0	0/0	1/0	2/0
Grade ≥ 3 AEs reported in > 1 patients with best response of SD								
Neutropenia	7/0	4/0	3/0	2/0	1/0	1/0	0/0	2/0
Thrombocytopenia	6/0	3/0	1/0	1/0	1/0	1/0	1/0	0/0
Infections SOC	2/3	1/2	0/0	0/0	0/0	0/0	1/0	0/2
Anemia	3/0	0/0	1/0	0/0	0/0	0/0	1/0	1/0

Patients who experienced drug-related events may have also experienced non-drug-related events

*SOC* system organ class

Of the 32 patients with best response of SD, 20 patients (63 %) and 6 patients (19 %) had ≥1 dose interruption and/or dose reduction due to AEs, respectively. Five of 32 patients with best response of SD discontinued romidepsin due to the following AEs: ventricular extrasystoles and decreased T wave amplitude (probably drug related; cycle 1); neutropenia (drug related; cycle 3); acute angle-closure glaucoma (possibly drug related; cycle 4); pulmonary embolism, elevated C-reactive protein, and melanoma (only melanoma was possibly drug related; cycle 6); and pneumonia (not drug related; cycle 9).

## Discussion

Three HDAC inhibitors have been approved by the US Food and Drug Administration for the treatment of TCL: romidepsin for patients with CTCL who have received ≥1 prior systemic therapy and for patients with PTCL who have received ≥1 prior therapy, vorinostat for cutaneous manifestations in patients with CTCL who have progressive, persistent, or recurrent disease on or following two systemic therapies, and belinostat for patients with relapsed or refractory PTCL [16, 20, 21]. In a pivotal phase 2 trial of patients with relapsed/refractory PTCL, single-agent belinostat was able to induce an ORR of 26 % (31/120 patients), including 11 % with complete response, and a DOR of 14 months. The median PFS and OS were 1.6 and 7.9 months, respectively, and common (>5 %) grade 3/4 adverse events included anemia (11 %), thrombocytopenia (7 %), dyspnea (6 %), and neutropenia (6 %) [22].

Single-agent romidepsin has been shown to lead to durable responses (median DOR >2 years [18]) in patients with relapsed/refractory PTCL regardless of baseline demographic and disease characteristics, including age, PTCL subtype, number or types of prior therapies, and response to prior therapy [17, 18]. The majority of responses (33/130; 25 % ORR) in the pivotal study were noted at the first response assessment (during cycle 2), and all were noted within the predetermined trial length of 6 cycles [18]. Although the NCCN guidelines for relapsed/refractory PTCL consider SD as a lack of response to treatment and a trigger to switch therapy [8], the protocol for the pivotal study of romidepsin allowed for continued treatment in patients with SD at the discretion of the patient and investigator. Thirty-two of the 130 patients (25 %) who did not achieve an objective response experienced disease stabilization, with most (22/32 (69 %)) achieving SD90 (response assessed as SD during cycles 2 and 4). Prolonged disease stabilization does not appear to be a result of inadequate response assessment, as the patients with SD ≥6 months by IRC assessment also had best response of SD by exploratory PET endpoint.

Although only six patients (19 %) achieved disease stabilization for ≥6 months, outcomes in terms of PFS and OS were similar for patients achieving PR or SD90. Rates of disease control (CR/CRu + PR + SD90) were 49, 44, and 38 for patients with PTCL-NOS, AITL, and ALK-1-negative ALCL, respectively. As a result of extended treatment in many patients, the pivotal study protocol was amended to allow for maintenance dosing after ≥12 treatment cycles.

It was previously reported that prolonged treatment with romidepsin did not affect the safety profile, and the highest incidence of grade ≥3 AEs occurred during cycles 1 to 2 of treatment [18]. Additionally, most discontinuations and dose modification occurred early in treatment (cycles 1 to 2 and 2 to 3, respectively). The most common AEs reported included gastrointestinal disturbances, hematologic abnormalities, asthenic conditions, and infections (all types pooled) [16–18]. There were no clinically significant changes in QT intervals across treatment cycles, and ECG abnormalities were uncommon. A recently published ECG study of romidepsin asserted the cardiac safety of romidepsin while stressing the need for appropriate potassium and/or magnesium supplementation throughout treatment [23]. Results from a thorough post-marketing cardiac study in patients with advanced malignancies also assert that despite the use of QT-prolonging antiemetics, romidepsin treatment did not significantly prolong QTc, even at supratherapeutic doses; reported increases in calculated QTc were exaggerated due to transient heart rate increases [24]. The AE profile for patients with best response of SD was similar to that of the overall population. Although the majority of patients with best response of SD experienced grade ≥3 AEs, <20 % required dose reductions and/or discontinued due to AEs. Patients with SD who received prolonged romidepsin treatment did experience AEs late in treatment, but only one patient discontinued due to AEs after cycle 6.

## Conclusions

The data shown herein demonstrated the feasibility and clinical benefit of prolonged administration of romidepsin in patients who achieved at least SD on therapy. Durability of responses in patients with relapsed/refractory PTCL remains a challenge with current treatment options [2, 4, 14], and both response rates and survival appear to decrease with increasing lines of prior therapy [15]. Durability of both objective responses and SD as well as long-term tolerability reported with romidepsin also warrants further investigation in studies of maintenance therapy after first-line chemotherapy or stem cell transplant in patients with PTCL.

## Methods

### Study design

The study design and eligibility criteria for this prospective, single-arm, open-label, international phase 2 study were previously described (Clinicaltrials.gov identifier: NCT00426764) [17]. The following PTCL subtypes [25] were eligible: PTCL-NOS, AITL, extranodal NK/TCL nasal type, enteropathy-type TCL, subcutaneous panniculitis-like TCL, cutaneous γδ TCL, hepatosplenic TCL, ALK-1-negative ALCL, ALK-1-positive ALCL (restricted to patients with disease relapse post autologous stem cell transplant), and transformed mycosis fungoides (nontransformed mycosis fungoides and Sézary syndrome excluded). Diagnosis of PTCL was histologically confirmed by local pathologists, and PTCL subtyping was reviewed by a central laboratory (Celligent Diagnostics, Charlotte, NC). Briefly, eligible patients had PTCL relapsed or refractory to ≥1 systemic therapy with measurable disease according to International Working Group (IWG) criteria [26] and/or measurable cutaneous disease, an Eastern Cooperative Oncology Group (ECOG) performance status of 0 to 2 at enrollment, adequate bone marrow and organ function (including no known significant cardiac abnormalities), and serum potassium and magnesium concentrations ≥3.8 and ≥0.85 mmol/L, respectively. The need for electrolyte supplementation is common for patients with TCL [23, 27], and hypokalemia and/or hypomagnesemia are known risk factors for cardiac arrhythmia and sudden cardiac death [28–31] and may be associated with electrocardiogram (ECG) abnormalities [32, 33]. Low levels of potassium and/or magnesium could be corrected by supplementation to meet the inclusion criteria throughout the trial.

Patients received romidepsin 14 mg/m^2^ as a 4-h intravenous infusion on days 1, 8, and 15 of each 28-day cycle (Food and Drug Administration-approved dosing in both PTCL and CTCL [16]) for up to 6 cycles. Patients with at least SD could continue treatment beyond 6 cycles at the discretion of the patient and investigator. By protocol amendment, patients treated for ≥12 cycles could receive maintenance dosing of two rather than three doses per 28-day cycle. After cycle 24, dosing could be further reduced to once per cycle in those who has received maintenance dosing for ≥6 months.

The protocol, informed consent form, and other relevant study documentation were approved by the institutional review boards of all participating institutions. All patients gave written informed consent prior to study entry.

### Efficacy and safety assessments

The efficacy and safety assessments conducted were previously described in detail [17]. Response was assessed every 2 cycles (during days 22–28, completed prior to treatment in next cycle) by site investigators and an independent review committee (IRC) according to the 1999 IWG criteria guidelines for response assessments for NHL [26]. IRC assessments were primary and investigator assessments were considered supportive. The primary endpoint was rate of CR/CRu, and key secondary efficacy endpoints included ORR, DOR, and time to disease progression. Time to response as well as survival (PFS and overall survival [OS]) by best response to romidepsin at any time on trial was also assessed. The utility of positron emission tomography (PET) in this patient population was examined as a prospective exploratory endpoint, with responses assessed by IWG + PET criteria [34]. Full results for this endpoint were presented in a separate publication [35].

In the analyses herein, SD90 was defined as patients with the best response of SD with time to progression ≥90 days, which corresponded with a response assessment of SD during cycles 2 and 4. Adverse events (AEs) were documented according to the Medical Dictionary for Regulatory Activities (version 12.0) and the National Cancer Institute Common Terminology Criteria for Adverse Events (version 3.0). Drug-related AEs were those indicated by the investigator as having at least a possible relationship to romidepsin or missing a relationship assessment.

### Statistical methods

Patients with the best response of SD were the primary focus of this analysis. All descriptive statistical analyses were performed using SAS statistical software, version 9.2 (SAS Institute). Time-to-event data were summarized by Kaplan-Meier methods. This study is ongoing, but September 30, 2012, was the cutoff date for this analysis. Patients who withdrew from the trial without PD were to be assessed every 2 months until PD, at withdrawal from follow-up, or at start of alternate therapy.
