# Erratum to: Romidepsin for the treatment of relapsed/refractory peripheral T cell lymphoma: prolonged stable disease provides clinical benefits for patients in the pivotal trial

**DOI:** 10.1186/s13045-017-0518-8

**Published:** 2017-09-18

**Authors:** Francine Foss, Steven Horwitz, Barbara Pro, H. Miles Prince, Lubomir Sokol, Barbara Balser, Julie Wolfson, Bertrand Coiffier

**Affiliations:** 1 Yale Cancer Center, 333 Cedar St, TMP 3, PO Box 208028, New Haven, CT 06520-8028 USA; 2 Memorial Sloan-Kettering Cancer Center, New York, NY USA; 3 Kimmel Cancer Center, Thomas Jefferson University, Philadelphia, PA USA; 4 Peter MacCallum Cancer Centre, University of Melbourne, Melbourne, Australia; 5 Moffitt Cancer Center, Tampa, FL USA; 6 Veristat, LLC, Southborough, MA USA; 7 Hospices Civils de Lyon, Lyon, France

## Erratum

The original article [1] contains an error whereby Fig. 2a & b are mistakenly interchanged and contain incorrect colour-coding.

**Fig. 2:**
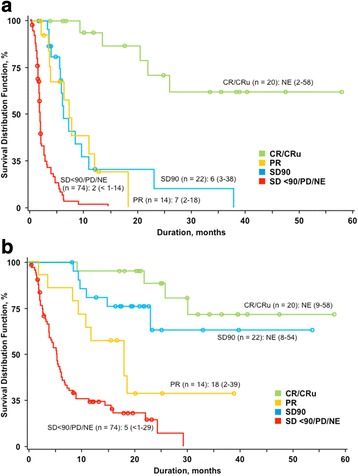
Survival based on clinical IRC assessment by best response to romidepsin (*n* = 130). Progression-free survival (**a**) and overall survival (**b**). Patients with insufficient efficacy data to determine response due to early termination (NE; *n* = 29) were included as nonresponders. *NE* not evaluable

The correct versions of Fig. 2a & b are thus displayed below.

## References

[1] Foss F (2016). Romidepsin for the treatment of relapsed/refractory peripheral T cell lymphoma: prolonged stable disease provides clinical benefits for patients in the pivotal trial. J Hematol Oncol.

